# Osmolyte accumulation regulates the SUMOylation and inclusion dynamics of the prionogenic Cyc8-Tup1 transcription corepressor

**DOI:** 10.1371/journal.pgen.1008115

**Published:** 2019-04-22

**Authors:** Cory M. Nadel, Timothy D. Mackie, Richard G. Gardner

**Affiliations:** Department of Pharmacology, University of Washington, Seattle, WA, United States of America; University of Massachusetts Amherst, UNITED STATES

## Abstract

Environmental stressors can severely perturb cellular homeostasis and compromise viability. To cope with environmental stressors, eukaryotes have developed distinct signaling programs that allow for adaptation during different stress conditions. These programs often require a host of post-translational modifications that alter proteins to elicit appropriate cellular responses. One crucial protein modifier during stress is the small ubiquitin-like modifier SUMO. In many cases, however, the functions of stress dependent protein SUMOylation remain unclear. Previously, we showed that the conserved *Saccharomyces cerevisiae* Cyc8-Tup1 transcriptional corepressor complex undergoes transient hyperosmotic stress-induced SUMOylation and inclusion formation, which are important for appropriate regulation of hyperosmotic-stress genes. Here, we show the osmostress-responsive MAP kinase Hog1 regulates Cyc8 SUMOylation and inclusion formation via its role in the transcriptional activation of glycerol biosynthesis genes. Mutations that ablate Cyc8 SUMOylation can partially rescue the osmosensitivity of *hog1Δ* cells, and this is facilitated by inappropriate derepression of glycerol-biosynthesis genes. Furthermore, cells specifically unable to synthesize the osmolyte glycerol cause transient Cyc8 SUMOylation and inclusions to persist, indicating a regulatory role for glycerol to reestablish the basal state of Cyc8 following adaptation to hyperosmotic stress. These observations unveil a novel intersection between phosphorylation and SUMOylation networks, which are critical for shifting gene expression and metabolic programs during stress adaptation.

## Author summary

The ability to sense and react to diverse environmental cues is a central aspect in the maintenance of cellular homeostasis. In response to harsh conditions, cells must rapidly deploy specific stress responses in order to adapt, survive, and proliferate. To ensure optimal spatial and temporal control over stress responses, many proteins undergo biophysical and biochemical alterations. More specifically, these alterations include conformational changes and post-translational modifications–such as phosphorylation, ubiquitination, and SUMOylation–that alter the function, localization, and interactome of target proteins. In this study, we show that the Hog1 MAPK regulates SUMOylation and biomolecular condensation of the yeast transcription corepressor complex Cyc8-Tup1 during exposure to hyperosmotic stress. In turn, this signaling relationship functions to effectively rewire yeast metabolism toward the biosynthesis of the compatible osmolyte glycerol, which serves as the ultimate signal to reset this genetic circuit.

## Introduction

Cellular stresses are abiotic perturbations that can severely and irreversibly damage biomolecules and essential cell structures. All organisms experience cellular stress and subsequently must adjust a variety of cellular programs to reestablish homeostasis. Many of these include signal transduction networks, metabolic pathways, gene expression programs, cell-cycle progression, and protein quality control systems. Resiliency in the face of stress is crucial to cellular survival with the deterioration of adaptive measures thought to underlie a variety of age-related human diseases such as cancer, heart disease, and neurodegeneration [1–3].

Post-translational protein modifications are critical for the responses to cellular stress, and often occur rapidly upon the onset of stress. One of the major modifications that have been observed to occur during a wide range of cellular stresses is the addition of the small-ubiquitin-like-modifier (SUMO) to proteins, with many of the targets of SUMOylation being specifically modified during distinct stress conditions [4, 5]. While many studies have documented the targets of stress-dependent SUMOylation, consequences of many SUMOylation events still remain poorly characterized [6]. Furthermore, it is still unclear what cellular factors regulate the dynamics of stress-dependent SUMOylation during cellular adaptation [7].

In the budding yeast *Saccharomyces cerevisiae*, exposure to hyperosmotic stress initiates a rapid transient wave of SUMOylation [8–10]. Yeast adaptation to hyperosmotic conditions is largely facilitated by the high osmolarity glycerol (HOG) signaling pathway, which converges on the yeast orthologue of mammalian p38 mitogen-activated protein kinase (MAPK), Hog1 [11, 12]. Upon activation, Hog1 initiates a multifaceted adaptive program that results in alterations in gene expression, temporary cell-cycle arrest, and a metabolic shift toward the synthesis and retention of the intracellular osmolyte glycerol [11]. Furthermore, loss of Hog1 function extends the duration of hyperosmotic stress-induced SUMOylation [8]. However, the targets of this extended SUMOylation and its functional purpose have yet to be understood.

We previously discovered that the primary targets of hyperosmotic stress-induced SUMOylation are the transcriptional corepressor proteins Tup1 and Cyc8 [10]. These proteins are rapidly and transiently SUMOylated following exposure to hyperosmotic conditions, and mutations that limit their SUMOylation drastically alter transcriptional patterns during adaptation to stress. Interestingly, the Cyc8-Tup1 complex forms reversible nuclear inclusions upon hyperosmotic shock, and the persistence of these inclusions correlates tightly with the duration of SUMOylation and the loss of transcriptional repression. Self-association of the Cyc8-Tup1 complex into inclusions is driven in large part by a disordered prion domain in Cyc8 [10], which is highly glutamine-rich [13]. Moreover, Cyc8 can be induced to form and propagate as a prion, and cells bearing the prion form of Cyc8 show derepression of target genes consistent with a partial loss-of-function phenotype [14]. Recent studies have shown that a variety of disordered or prionogenic proteins form dynamic inclusion bodies under stress conditions, but it remains unclear what facilitates a shift from soluble, liquid-like states to insoluble, solid-like states [15, 16]. Here, we explore how Cyc8 inclusions and SUMOylation are modulated by the Hog1-dependent accumulation of the intracellular osmolyte glycerol during hyperosmotic stress.

## Results

### The Hog1 MAPK regulates the persistence of Cyc8-Tup1 SUMOylation

**Fig 1:**
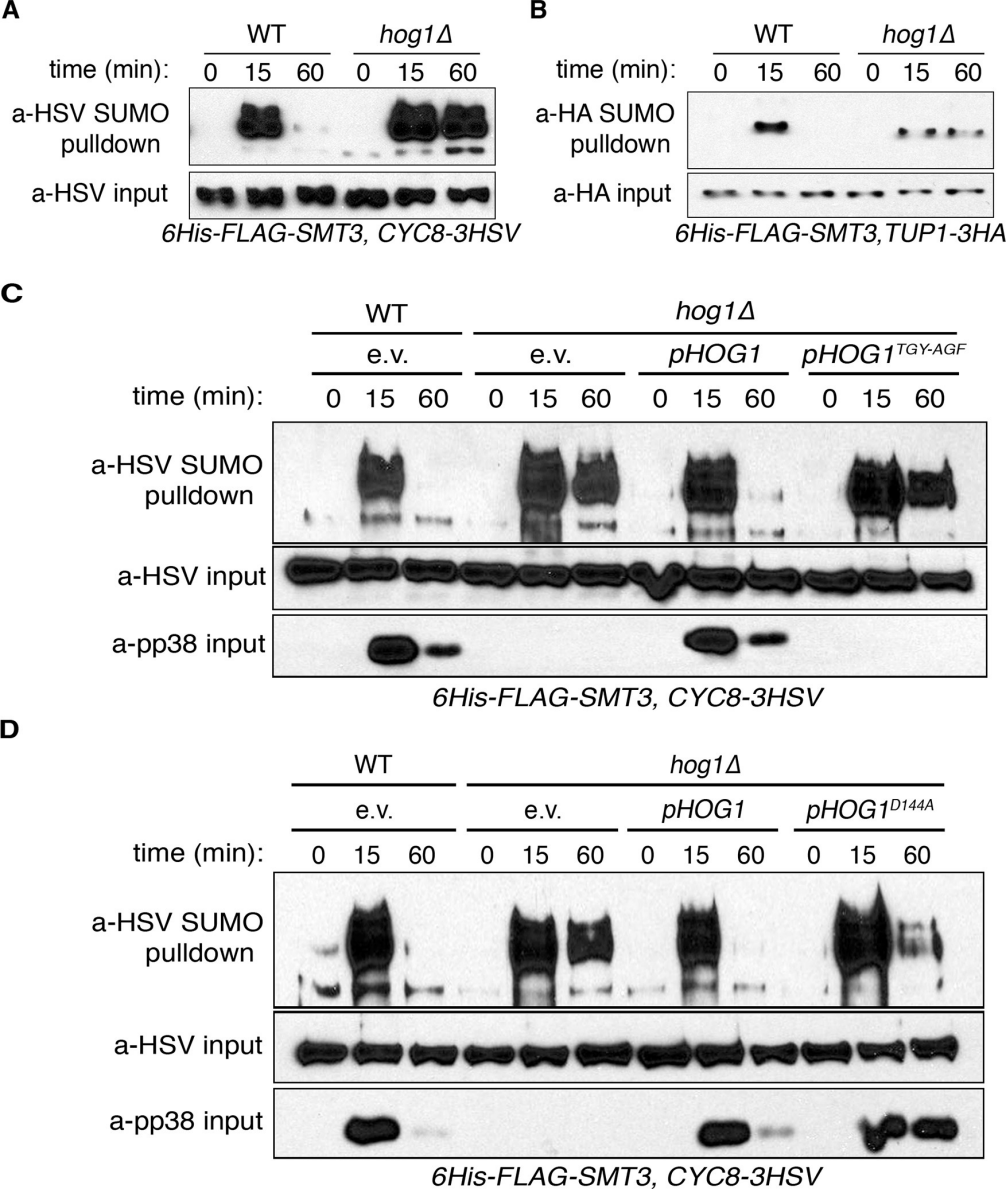
The Hog1 MAPK regulates the duration of Cyc8-Tup1 SUMOylation. (A) Examination of Cyc8 SUMOylation after exposure to hyperosmotic stress. Parent or *hog1Δ* cells expressing 6His-FLAG-Smt3 and Cyc8-3HSV were treated with 1.2M sorbitol, collected at the indicated time points, and SUMOylated proteins were isolated by metal affinity chromatography. Proteins were separated by SDS-PAGE and Cyc8 was identified by Western analysis using anti-HSV antibodies. Total Cyc8 in the input fraction was used as a loading control. (B) Examination of Tup1 SUMOylation after exposure to hyperosmotic stress. Parent or *hog1Δ* cells expressing 6His-FLAG-Smt3 and Tup1-3HA were treated with 1.2M sorbitol, collected at the indicated time points, and SUMOylated proteins were isolated by metal affinity chromatography. Proteins were separated by SDS-PAGE and Tup1 was identified by Western analysis using anti-HA antibodies. Total Tup1 in the input fraction was used as a loading control. (C) Maintenance of normal Cyc8 SUMOylation requires Hog1 activation. Parent or *hog1Δ* cells expressing the indicated constructs, 6His-FLAG-Smt3, and Cyc8-3HSV were treated, collected, and analyzed as described in Fig 1A. Cyc8 was identified by Western analysis using anti-HSV antibodies. Total Cyc8 in the input fraction was used as a loading control, while activated p38 was used as a control for Hog1 presence and activation. (D) Maintenance of normal Cyc8 SUMOylation requires Hog1 catalytic activity. Parent or hog1*Δ* cells expressing the indicated constructs, 6His-FLAG-Smt3, and Cyc8-3HSV were treated, collected, and analyzed as described in Fig 1A. Cyc8 was identified by Western analysis using anti-HSV antibodies. Total Cyc8 in the input fraction was used as a loading control, while activated p38 was used as a control for Hog1 presence and activation.

Hog1 has been shown to limit the accumulation of high molecular weight SUMO conjugates after the induction of hyperosmotic stress [8, 10], but the proteins that comprise the Hog1-regulated SUMO conjugates had not been identified. We previously demonstrated that the Cyc8-Tup1 corepressor complex comprised the major targets of SUMOylation following hyperosmotic stress exposure [10]. Given this, we hypothesized that Hog1 regulates the duration of Cyc8-Tup1 SUMOylation following exposure to hyperosmotic stress. To test this, we performed metal affinity purification of SUMOylated proteins on yeast cells expressing modified version of the yeast SUMO protein, His_6_-FLAG-Smt3, from the endogenous *SMT3* promoter after exposure to 1.2M sorbitol. We followed the purification by examining the SUMOylation state for Cyc8 or Tup1 via Western analysis. While parent cells showed transient SUMOylation of both Cyc8 and Tup1, *hog1Δ* cells showed prolonged SUMOylation of both complex members, with the SUMOylation persisting to at least 60 minutes post stress induction (Fig 1A and 1B, WT vs *hog1Δ*).

Hog1 function depends on both phosphorylation-dependent activation using an established *TXY* motif and catalytic function through a defined kinase domain [17]. Because Cyc8 SUMOylation is the key initiating event for SUMOylation of the Cyc8-Tup1 complex [10], we wanted to verify that these individual Hog1 functions were necessary for the regulation of Cyc8 SUMOylation. To do so, we generated plasmids encoding intact (WT), activation-deficient (TGY-AGA), or kinase-deficient (D144A) Hog1 and expressed them ectopically in *hog1Δ* cells. The hyperosmotic sensitivity of the mutants was measured by a spot titer assay on synthetic medium containing an elevated concentration of KCl (S1 Fig). Each *hog1* mutant strain showed the same sensitivity as the *hog1Δ* strain. To assess the effect of the *hog1* mutations on Cyc8 SUMOylation, SUMOylated proteins were purified from these strains by metal affinity chromatography before and after exposure to 1.2M sorbitol, and Cyc8 SUMOylation was examined by Western analysis. While ectopic expression of WT Hog1 rescued the delay in Cyc8 deSUMOylation, both activation-dead and kinase-dead Hog1 showed prolonged Cyc8 SUMOylation similar to that of the complete *HOG1* gene deletion (Fig 1C and 1D).

Upon exposure to hyperosmotic stress, Hog1 feedback can inhibit the pheromone response MAPK cascade to prevent inappropriate activation of the mating pathway, which shares the upstream MAPKKK Ste11 [11]. As a result, *hog1Δ* cells can undergo inappropriate activation of the mating pathway upon exposure to hyperosmotic stress. To ensure that our observations on Cyc8 SUMOylation were not due to crosstalk, we generated *ste11Δ* cells and examined the distribution of high molecular weight SUMO conjugates by Western analysis. Compared to the prolonged SUMOylation observed in *hog1Δ* cells, *ste11Δ* cells showed the same transient SUMOylation profile as parent cells, indicating that crosstalk with the mating pathway was not responsible for the delay in Cyc8 deSUMOylation (S1 Fig).

### Active transcription is required for the transient dynamics of Cyc8 SUMOylation and inclusion formation during hyperosmotic stress

**Fig 2:**
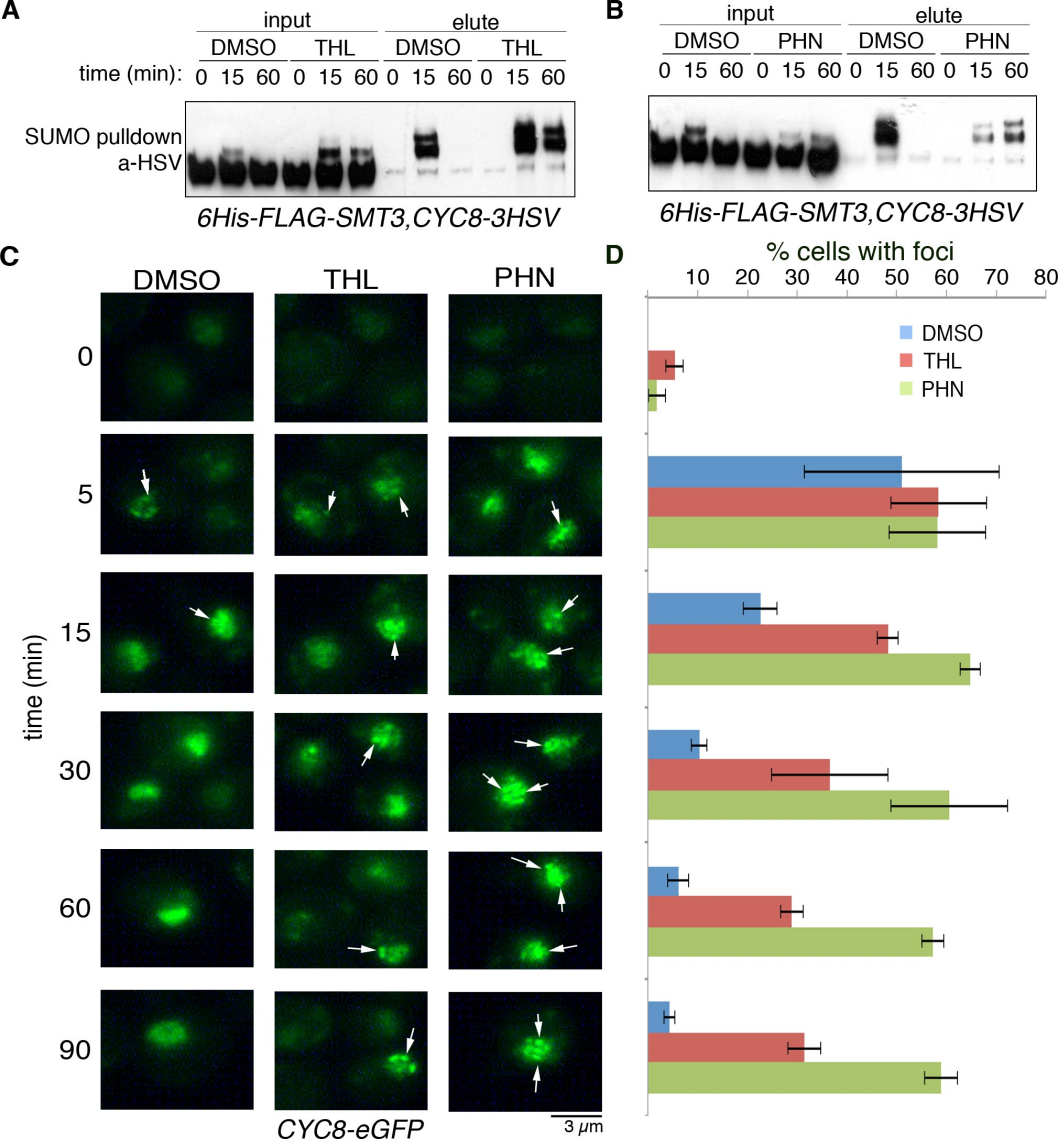
Cyc8 SUMOylation and focal localization are persistent in the absence of active transcription. (A) Thiolutin (THL) prolongs Cyc8 SUMOylation during hyperosmotic stress. Parent cells expressing 6His-FLAG-Smt3 and Cyc8-3HSV were grown in complete synthetic medium, treated with 4ug/ml THL or DMSO vehicle control for 5 minutes, and then exposed to 1.2M sorbitol. Cells were collected and analyzed as described in Fig 1A. Cyc8 was identified by Western analysis using anti-HSV antibodies. Total Cyc8 in the input fraction was used as loading control. (B) 1,10-phenanthroline (PHN) prolongs Cyc8 SUMOylation during hyperosmotic stress. Parent cells expressing 6His-FLAG-Smc3 and Cyc8-3HSV were grown in complete synthetic medium, treated with 500ug/ml PHN or DMSO vehicle control for 5 minutes, and then exposed to 1.2M sorbitol. Cells were collected and analyzed as described in Fig 1A. Cyc8 was identified by Western analysis using anti-HSV antibodies. Total Cyc8 in the input fraction was used as loading control. (C) Comparison of Cyc8 inclusions lifetime after transcription inhibition during exposure to hyperosmotic stress. Parent cells expressing Cyc8-eGFP were grown in complete synthetic medium and treated with the indicated compound for 5 minutes at room temperature. Cells were then treated with 1.2M sorbitol, collected by centrifugation, and fixed in 4% paraformaldehyde at the denoted time points. Cells were washed in PBS, spotted onto glass slides, and Cyc8 was imaged by fluorescence microscopy. (D) Quantification of cells bearing Cyc8 inclusions. Inclusions-bearing cells from Fig 2C were counted and represented as percentage of total cells. Error bars show SD.

Hog1 functions in the hyperosmotic stress response by altering transcription, translation, and post-translation programs in the cell [11]. Because Cyc8 is a transcription corepressor, we wanted to determine whether the accumulation and/or persistence of SUMOylated Cyc8 following hyperosmotic stress was regulated by active transcription. We chose pharmacological manipulation because that allows for transcription to be inhibited rapidly and acutely. To do this, we used the transcription inhibitors thiolutin (THL) and 1,10-phenanthroline (PHN). These drugs block transcription through distinct mechanisms: THL directly inhibits RNA polymerase while PHN is a potent chelator of metal ions that function as cofactors for RNA polymerases [18, 19]. We found that acute pretreatment of cells with both drugs prolonged Cyc8 SUMOylation following the onset of hyperosmotic stress. In both cases after pretreatment, Cyc8 SUMOylation was maintained at least to 60 minutes following hyperosmotic stress induction (Fig 2A and 2B). While both drugs prolonged the duration of Cyc8 SUMOylation, the relative levels of hyperosmotic stress-induced Cyc8 SUMOylation were different: THL pretreatment showed initial Cyc8 SUMOylation equivalent to vehicle pretreatment, whereas PHN pretreatment showed initial Cyc8 SUMOylation much less than vehicle treatment. As stated previously, PHN is a metal chelator that forms stable complexes with a variety of divalent metals including Zn^2+^. SUMO ligases use Zn^2+^ as an essential cofactor [20]. Thus, the lower levels of initial Cyc8 SUMOylation seen after PHN pretreatment prior to hyperosmotic stress induction likely reflects inhibition of SUMO ligase activity, which would explain the observed differences in relative SUMOylation following THL and PHN pretreatment.

We previously reported that Cyc8 forms transient inclusions during hyperosmotic stress that correlated with Cyc8 SUMOylation kinetics and dynamics [10]. As such, we hypothesized that transcription inhibition that prolonged SUMOylation of Cyc8 would also prolong the persistence of Cyc8 inclusions. To test this, we pretreated parent cells expressing Cyc8-eGFP from the endogenous *CYC8* locus with vehicle, THL, or PHN, challenged them with hyperosmotic stress, and imaged by fluorescence microscopy (Fig 2C). Treatment with either THL or PHN significantly prolonged the lifetime of Cyc8 nuclear inclusions during hyperosmotic stress (Fig 2D). Thus, both transient Cyc8 SUMOylation and inclusion kinetics/dynamics require active transcription, suggesting that a regulatory factor must be produced through gene expression to elicit appropriate Cyc8 SUMOylation and behavior during hyperosmotic stress.

### Loss of Cyc8 SUMOylation partially suppresses the osmosensitivity of *hog1Δ* cells.

**Fig 3:**
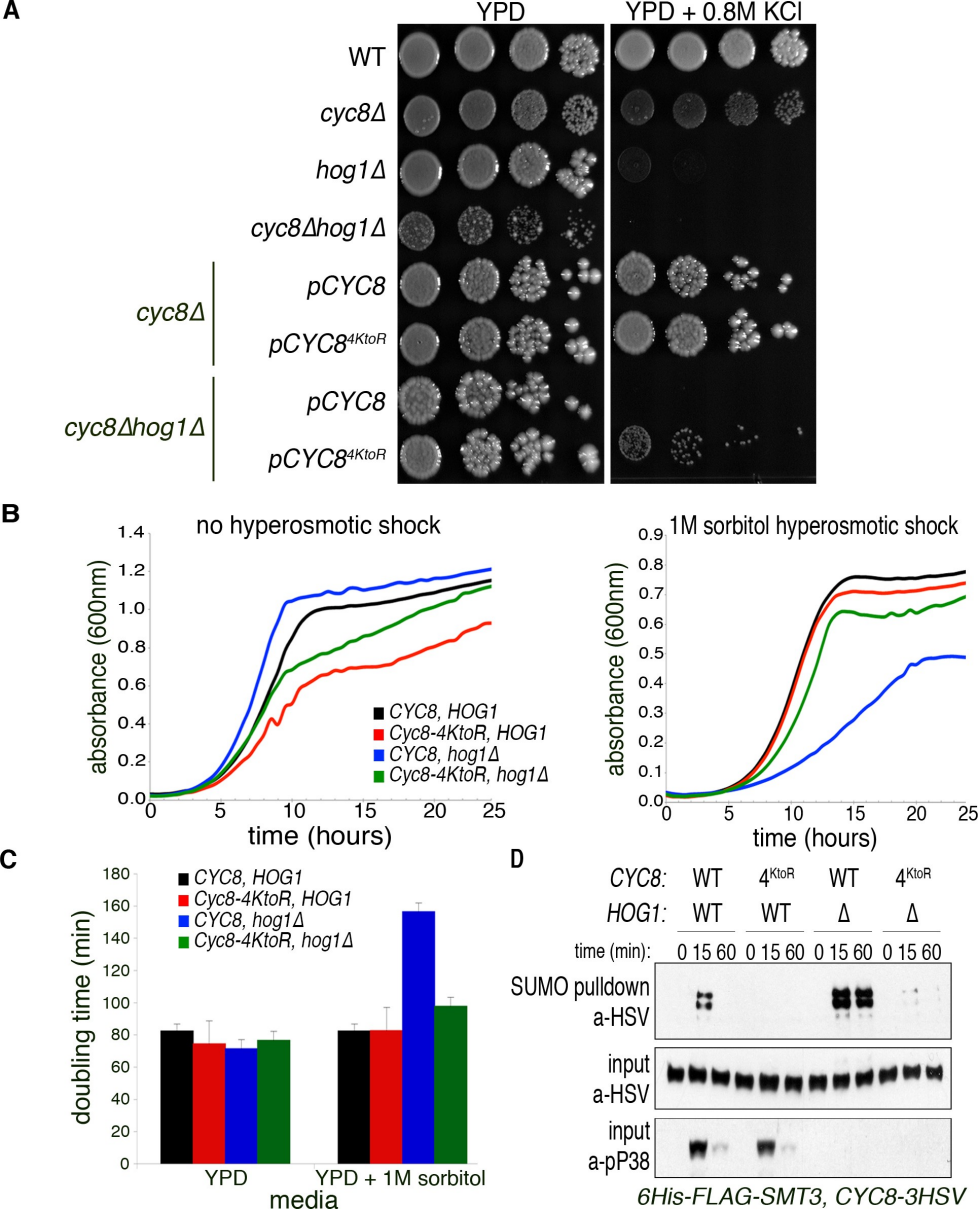
Loss of Cyc8 SUMOylation suppresses the osmosensitivity of hog1*Δ* cells (A) Spot titer assay comparing osmosensitivity of various cells. Parent, *cyc8Δ*, *hog1Δ*, or *cyc8Δhog1Δ* yeast cells were transformed with the indicated constructs and spotted in ten-fold serial dilutions on YPD or YPD+0.8MKCl plates and grown at 30°C for two days. (B) Quantitative measure of growth rates generated by Bioscreen C. Indicated cells were grown in triplicate at 30°C in YPD or YPD+1M sorbitol for 24 hours with continuous shaking. Absorbance at 600nm was measured every 30 minutes and average absorbance was plotted versus time. (C) Average doubling times in specific media as measured by Bioscreen C. Doubling times for indicated cells were calculated using the yeast outgrowth data analyzer (YODA, [22]). Error bars show SD for triplicate samples. (D) Confirmation of reduced Cyc8 SUMOylation in suppressor cells. Indicated cells bearing 6His-FLAG-Smc3 and 3HSV-tagged Cyc8 were treated with 1.2M sorbitol and collected at the indicated time points. SUMOylated proteins were collected and analyzed as described in Fig 1A. SUMOylated-Cyc8 was identified by Western analysis using anti-HSV antibodies. Total Cyc8 in the input fraction was used as a loading control, while activated p38 was used as a control for Hog1 presence and activation.

Cyc8 SUMOylation was prolonged when transcription was inhibited, so we explored the consequences of eliminating Cyc8 SUMOylation. We previously revealed that hyperosmotic stress-dependent Cyc8 SUMOylation primarily occurs at a cluster of four lysine residues (position 735, 736, 738, and 748) located C-terminal to the TPR region [10]. We created four Lys-to-Arg mutations that ablate SUMOylation of Cyc8, which we call Cyc8^4KtoR^. These mutations reside in the Cyc8 C-terminal region that, when deleted in its entirety, partially suppressed the osmosensitivity of *hog1Δ* cells, and was correlated with derepression of hyperosmotic stress-responsive genes [21]. Because this large deletion, from residues 389–966, may contain other important regulatory regions, we asked if only the mutation of the Cyc8 SUMOylation sites could suppress the osmosensitivity of *hog1Δ* cells using a spot titer assay. While mutation of the Cyc8 SUMOylation sites did not alter growth of *HOG1* cells, loss of Cyc8 SUMOylation partially suppressed the osmosensitivity of *hog1Δ* cells (Fig 3A), similar to previous studies using the Cyc8 C-terminal truncation mutant. We verified the appropriate reduction of Cyc8 SUMOylation in Cyc8^4KtoR^ mutant cells by performing metal affinity purification of SUMOylated proteins and probing for Cyc8-3HSV by Western analysis (Fig 3D).

Spot titer assays do not reveal quantitative growth rates. Therefore, we analyzed growth rates under hyperosmotic stress using a Bioscreen C automated growth curve analyzer (Fig 3B). When *HOG1* is present, the SUMOylation-deficient Cyc8^4KtoR^ had little effect on cells' ability to grow under stress. In *hog1Δ* cells, the presence of SUMOylation-deficient Cyc8^4KtoR^ accelerated the cells’ growth rate when compared to SUMOylation-proficient Cyc8. We calculated doubling times for all yeast strains using the Yeast Outgrowth Data Analyzer software [22], and found that loss of Cyc8 SUMOylation significantly reduced the relative doubling time of *hog1Δ* cells when grown during hyperosmotic stress (Fig 3C), similar to the spot titer assay. Altogether, loss of Cyc8 SUMOylation is sufficient to reestablish growth of *hog1Δ* cells in hyperosmotic conditions.

### Loss of Cyc8 SUMOylation in *hog1Δ* cells partially rescues hyperosmotic stress-induced glycerol biosynthesis

Loss of Cyc8 SUMOylation in *hog1Δ* cells partially restored the viability of *hog1Δ* cells during hyperosmotic stress. Because Cyc8 is a transcription corepressor that engages chromatin-bound transcription factors in target gene promoters, we wanted to examine the effects of the *hog1Δ* allele and loss of Cyc8 SUMOylation on Cyc8 chromatin occupancy. We chose to interrogate Cyc8 promoter occupancy at the glycerol biosynthetic enzyme *GPD1* gene for two reasons. First, while Hog1 activation by hyperosmotic stress upregulates the expression of >100 genes upon hyperosmotic stress [23], only the increased expression of *GPD1* is required for cellular survival during hyperosmotic stress via its role in the biogenesis of the intracellular osmolyte glycerol [24]. Second, *GPD1* is a known target of Cyc8-mediated repression under non-stress conditions [25]. We hypothesized that altered Cyc8 occupancy at *GPD1* when Cyc8 SUMOylation was ablated could lead to increased production of glycerol in *hog1Δ* cells.

**Fig 4:**
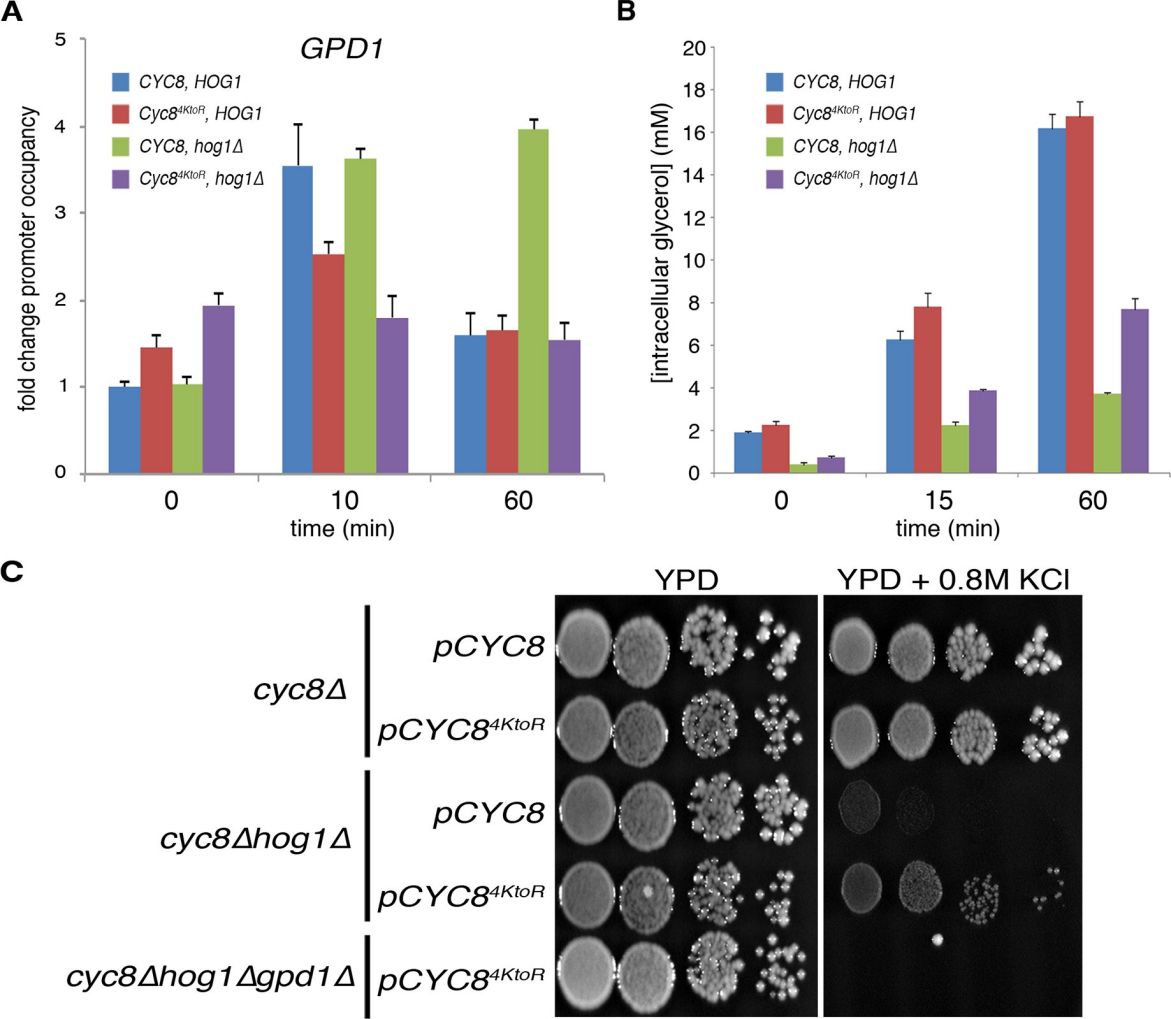
Loss of Cyc8 SUMOylation in *hog1Δ* cells reestablishes hyperosmotic stress-induced glycerol biosynthesis. (A) Cyc8 transiently associates with the *GPD1* promoter during hyperosmotic stress. Indicated cells were grown in triplicate in rich medium, treated with 1.2M sorbitol, and collected at the denoted time points. Cyc8-DNA complexes were crosslinked with formaldehyde, immunoprecipitated via incubation with anti-HSV antibody, and analyzed by qPCR with specific primers (sequences listed in Materials and Methods). Changes in Cyc8 promoter occupancy were corrected to *ACT1* and represented as fold change over pre-stress condition. Error bars show SD. (B) Glycerol accumulation assay after exposure to hyperosmotic stress. Indicated cells were grown in triplicate in rich medium, treated with 1.2M sorbitol, and collected at the denoted time points. Lysates were extracted by boiling at 95°C for 10 minutes in TBS, clarified by centrifugation, and glycerol was analyzed by colorimetric assay. Error bars show SD. (C) Spot titer assay comparing osmosensitivity of various cells. Indicated cells were spotted in ten-fold serial dilutions on YPD or YPD+0.8M KCl and grown at 30°C for two days.

To test this hypothesis, we examined Cyc8 or SUMOylation-deficient Cyc8^4KtoR^ association at the *GPD1* promoter using chromatin immunoprecipitation (ChIP) and quantitative PCR (ChIP-qPCR) in *HOG1* and *hog1Δ* cells (Fig 4A). In *HOG1* cells, Cyc8 became enriched at the *GPD1* promoter within 10 minutes of stress exposure and returned to baseline levels after 60 minutes. In *hog1Δ* cells, Cyc8 had nearly identical enrichment at the *GPD1* promoter prior to stress onset and during the initial phase of adaptation (10 minutes). However, Cyc8 continued to be highly enriched at the *GPD1* promoter 60 minutes after stress onset in *hog1Δ* cells. SUMOylation-deficient Cyc8^4KtoR^ showed different *GPD1* promoter occupancy that Cyc8. In *HOG1* cells, Cyc8^4KtoR^ had slightly increased *GPD1* promoter occupancy prior to the onset of stress. During the initial phase of stress exposure, Cyc8^4KtoR^ was enriched at the *GPD1* promoter, but it was reduced compared with Cyc8. After 60 minutes of stress exposure, Cyc8^4KtoR^ occupancy at the *GPD1* promoter returned to baseline, similar to Cyc8. In *hog1Δ* cells, Cyc8^4KtoR^ had greater *GPD1* promoter occupancy prior to the onset of stress. There was no further enrichment at the *GPD1* promoter for Cyc8^4KtoR^ in *hog1Δ* cells during the course of stress exposure.

We next examined whether Cyc8 SUMOylation deficiency correlated with alterations in glycerol production that might explain the partial suppression of osmosensitivity of *hog1Δ* cells. To do this, we generated lysates from the various cells across a time course of hyperosmotic stress and analyzed the levels of glycerol present by a colorimetric assay (Fig 4B). In cells with *HOG1* intact and either Cyc8 or Cyc8^4KtoR^, there was rapid, robust glycerol accumulation upon the onset of hyperosmotic stress. In *hog1Δ* cells, there was markedly reduced glycerol accumulation, but SUMOylation-deficient Cyc8^4KtoR^ partially rescued glycerol accumulation over the course of hyperosmotic stress. The accumulation of approximately 50% of glycerol in SUMOylation-deficient Cyc8^4KtoR^ *hog1Δ* cells during exposure to hyperosmotic stress is consistent with the partial suppression of osmosensitivity observed in Fig 3.

Although we observed elevated intracellular glycerol in *Cyc8*^*4KtoR*^*hog1Δ* cells, we wanted to ensure that glycerol biosynthesis was necessary for the observed suppression of osmosensitivity. Therefore, we deleted *GPD1* from *Cyc8*^*4KtoR*^ *hog1Δ* cells and compared growth of these cells to control cells under hyperosmotic stress conditions by spot titer assay (Fig 4C). The additional deletion of *GPD1* from *Cyc8*^*4KtoR*^ *hog1Δ* cells did not alter growth on normal media. However, *Cyc8*^*4KtoR*^*hog1Δgpd1Δ* cells were highly osmosensitive and now equivalent to *hog1Δ* cells. Taken together, we conclude that altered *GPD1* promoter occupancy of Cyc8^4KtoR^ can change the capacity to synthesize glycerol in the absence of Hog1, and this leads to the partial suppression of osmosensitivity in *hog1Δ* cells by Cyc8^4KtoR^.

### Glycerol biosynthesis regulates the kinetics of Cyc8 SUMOylation and inclusion formation

**Fig 5:**
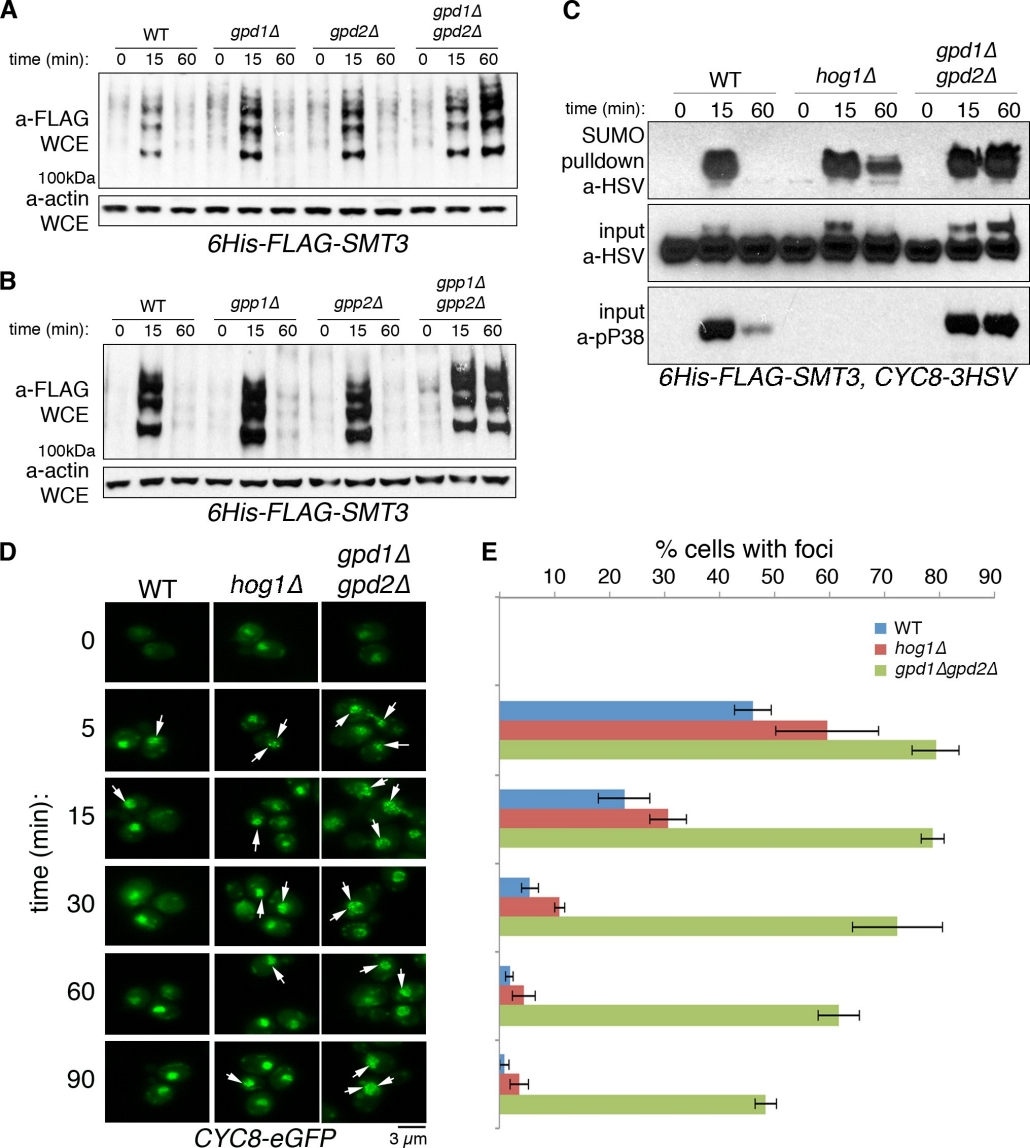
Cyc8 SUMOylation and inclusions persist in the absence of glycerol biosynthesis. (A) Combinatorial deletion of glycerol-3-phosphate dehydrogenases prolongs global SUMOylation during hyperosmotic stress. Indicated cells expressing 6His-FLAG-Smc3 were treated with 1.2M sorbitol and collected at the indicated time points. Whole cell extracts were separated by SDS-PAGE and Western analysis with anti-FLAG antibodies to identify SUMOylated proteins. Anti-actin antibodies were used to detect actin as a loading control. (B) Combinatorial deletion of glycerol-3-phosphate phosphatases prolongs global SUMOylation during hyperosmotic stress. Indicated cells were treated, collected, and analyzed as described in Fig 5A. (C) Cyc8 SUMOylation is prolonged in the absence of glycerol biosynthesis. Indicated cells treated, collected, and analyzed as described in Fig 1A. Cyc8 was identified by Western analysis using anti-HSV antibodies. Total Cyc8 in the input fraction was used as a loading control, while anti-pP38 was used as a control for Hog1 presence and activation. (D) Comparison of Cyc8 inclusions lifetime during exposure to hyperosmotic stress. Parent, *hog1Δ*, or *gpd1Δgpd2Δ* cells expressing Cyc8-eGFP were treated with 1.2M sorbitol, collected at the denoted timepoints, and fluorescence microscopy was performed as in Fig 3C (E) Quantification of cells bearing Cyc8 inclusions. Inclusions-bearing cells from Fig 5D were counted and represented as percentage of total cells. Error bars show SD.

Loss of Cyc8 SUMOylation partially rescued glycerol biosynthesis in *hog1Δ* cells. Therefore, we investigated if glycerol biosynthesis regulated Cyc8 SUMOylation. Glycerol biosynthesis is a two-step process in *S*. *cerevisiae*. First, dihydroxyacetone phosphate (DHAP) is reduced to glycerol-3-phosphate (G3P) in the rate-limiting step by the redundant glyceraldehyde-phosphate dehydrogenases 1 and 2 (Gpd1/Gpd2) [26]. Following, G3P is dephosphorylated by glycerol-phosphate phosphatases 1 and 2 (Gpp1/Gpp2) to yield glycerol [26]. To examine whether glycerol biosynthesis regulated hyperosmotic stress-induced SUMOylation, we deleted components of the glycerol biosynthesis machinery and analyzed global SUMOylation kinetics by Western analysis. We found that deletion of individual components of either step of glycerol biosynthesis did not alter SUMOylation kinetics from that of parent cells (Fig 5A). However, when the glycerol biosynthesis pathway was fully ablated by combinatorial deletion of *GPD1* and *GPD2*, hyperosmotic stress-induced SUMOylation was prolonged to at least 60 minutes after exposure. G3P is a necessary precursor to the production of lysophosphatidic acid (LPA), a phospholipid derivative with myriad cellular functions [27]. To verify that our findings were due to loss of glycerol and not of LPA, we generated cells deleted for the downstream glycerol biogenesis enzymes Gpp1 and Gpp2 (Fig 5B). As with the upstream Gpd proteins, only when both *GPP1* and *GPP2* were deleted did we prolong hyperosmotic stress-induced SUMOylation from that of parent cells. These data indicated that glycerol biosynthesis is a key regulator of hyperosmotic stress-induced SUMOylation.

To verify that the observed delay in deSUMOylation kinetics was specific to Cyc8, we purified SUMOylated proteins from parent, *hog1Δ*, and *gpd1Δgpd2Δ* cells by metal affinity purification and examined Cyc8 SUMOylation by Western analysis (Fig 5C). As seen before, parent cells show a transient pattern of Cyc8 SUMOylation wherein Cyc8 is transiently SUMOylated upon hyperosmotic stress. As seen before in Fig 1A, deletion of *HOG1* delays the deSUMOylation of Cyc8, resulting in a significant amount of Cyc8 remaining SUMOylated at 60 minutes in *hog1Δ* cells (Fig 5C). Cells that are *gpd1Δgpd2Δ* show robust SUMOylation of Cyc8 at 60 minutes, more so than in *hog1Δ* cells. It has been shown that *hog1Δ* cells accumulate glycerol under hyperosmotic stress, but do so at significantly slower rates than parent cells [28]. To illustrate the differences in glycerol accumulation between the different deletion cells, we performed colorimetric assays (S2 Fig). As expected, parent cells rapidly and robustly accumulated glycerol over the course of an hour under hyperosmotic stress, whereas *hog1Δ* cells accumulated glycerol slowly with a maximal accumulation of approximately 66% that of parent cells. By contrast, *gpd1Δgpd2Δ* cells accumulated virtually no glycerol over the observed time course. To verify the glycerol accumulation was due to active transcription, we treated parent cells with THL or PHN prior to exposure to hyperosmotic stress. These treatments significantly reduced glycerol accumulation (S2 Fig), further strengthening the hypothesis that stress-induced Cyc8 SUMOylation persists until glycerol has accumulated to sufficient levels and that active transcription of glycerol biosynthesis genes is necessary for the accumulation.

To establish that Cyc8 deSUMOylation is linked to glycerol biosynthesis and not to the action of glycerol activating another kinase signaling pathway, we chose to broadly inhibit intracellular kinases prior to hyperosmotic stress. We found that widespread kinase inhibition by pretreatment with the broad-spectrum kinase inhibitor staurosporine did not change Cyc8 SUMOylation dynamics during adaptation to hyperosmotic stress (S3 Fig). Importantly, we performed this experiment at a concentration of staurosporine found to inhibit PKA, but not Hog1 (S3 Fig). Thus, kinase pathways outside the Hog1 MAPK do not appear to be involved in regulating the dynamics of Cyc8 SUMOylation.

Due to our observation of prolonged Cyc8 SUMOylation in the absence of glycerol biosynthesis, we wanted to investigate whether glycerol accumulation regulated the resolution of Cyc8 inclusions. Using parent, *hog1Δ*, and *gpd1Δgpd2Δ* cells expressing Cyc8-eGFP, we performed fluorescence microscopy experiments across a time course of hyperosmotic stress (Fig 5D). As we observed previously, Cyc8 forms nuclear inclusions in parent cells that are resolved within 15 minutes. By contrast, *hog1Δ* cells show elevated prevalence of inclusions and slightly delayed resolution, with inclusions persisting to at least 30 minutes. In cells that are *gpd1Δgpd2Δ*, we observed robust inclusion formation with no observable resolution across the entire 90 minutes of observation. By quantifying the relative number of cells with Cyc8 inclusion for each background across the time course of stress, there was a significant increase in Cyc8 inclusion number and lifespan in the absence of glycerol accumulation (Fig 5E).

### Cyc8 nuclear inclusion resolution after transcriptional restart requires glycerol biosynthesis

**Fig 6:**
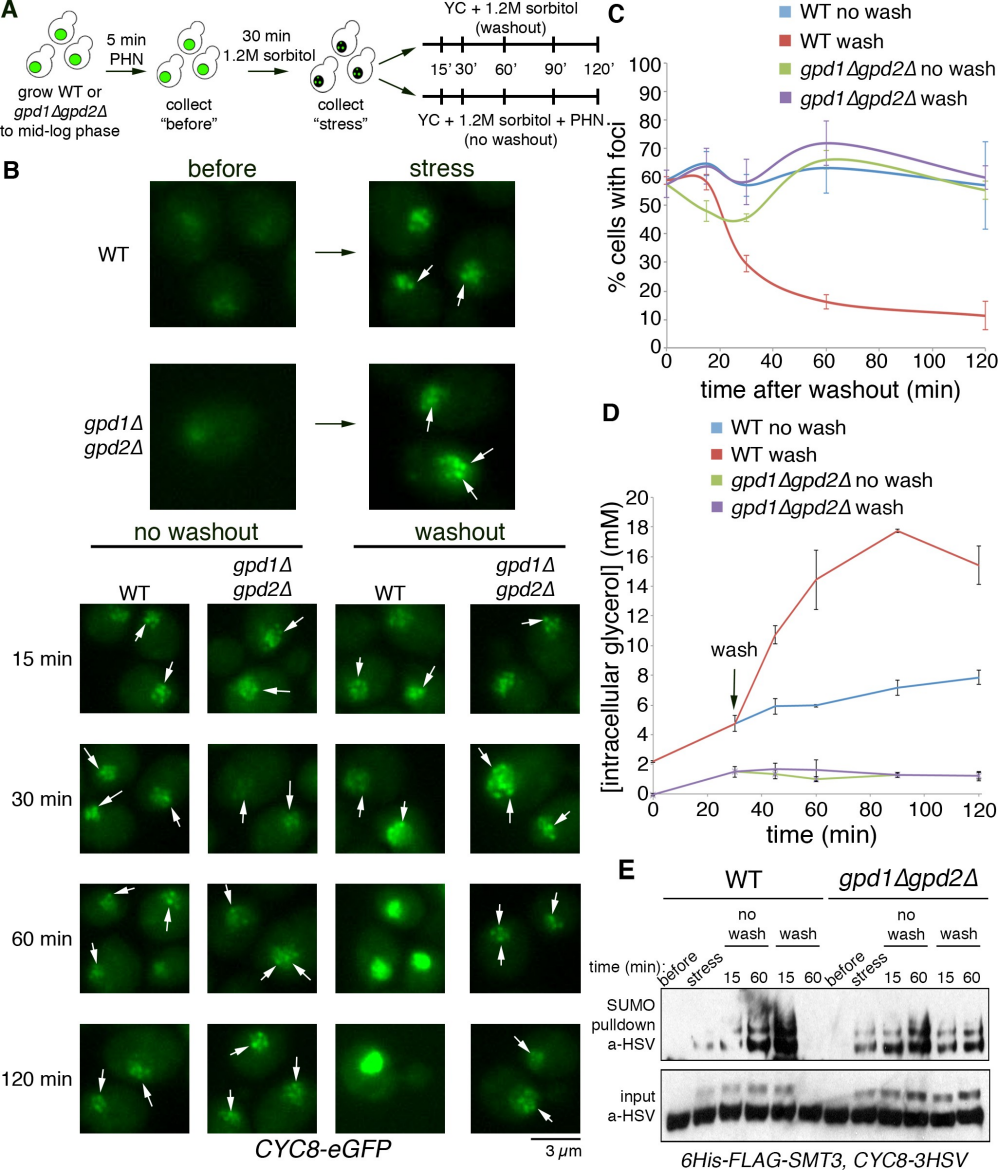
Cyc8 nuclear inclusions resolution after transcriptional restart requires glycerol biosynthesis. (A) Experimental design of PHN washout experiments. Parent or *gpd1Δgpd2Δ* cells were grown in complete synthetic medium, treated with 500 ug/ml PHN for 5 minutes, and then challenged with 1.2M sorbitol. Samples were collected prior to the onset of stress and following 30 minutes of hyperosmotic stress. Following, cells were collected by centrifugation and resuspended in fresh media containing 1.2M sorbitol with PHN (no wash) or with DMSO vehicle control (wash). Samples were collected at indicated timepoints following washout. (B) Comparison of Cyc8 inclusions lifetime after washout of PHN. Parent or *gpd1Δgpd2Δ* cells expressing Cyc8-eGFP were subjected to the experimental protocol described above. Cells were collected at the indicated time points following resuspension and fixed in 4% paraformaldehyde. Samples were then collected and fluorescence microscopy was performed as described in Fig 4E. (C) Quantification of cells bearing Cyc8 inclusions. Inclusion-bearing cells from Fig 7B were counted and represented as percentage of total cells. Error bars show SD. (D) Glycerol accumulation assay after removal of PHN. Parent or *gpd1Δgpd2Δ* cells expressing 6His-FLAG-Smt3 and Cyc8-3HSV were subjected to the experimental protocol described above. Lysates were extracted and glycerol content was analyzed as described in Fig 4B. Error bars show SD. (E) Comparison of Cyc8 SUMOylation after washout of PHN. Parent or *gpd1Δgpd2Δ* cells expressing 6His-FLAG-Smt3 and Cyc8-3HSV were subjected to the experimental protocol described above. SUMOylation was analyzed as described in Fig 1A. Cyc8 was identified by Western analysis using anti-HSV antibodies. Total Cyc8 in the input fraction was used as a loading control.

Both acute THL and PHN treatment prolonged Cyc8 inclusions (Fig 2C and 2D), and prevented glycerol accumulation (S2B Fig). However, it was unclear whether the action of these transcription inhibitors on glycerol biosynthesis was a causative reason for the extended persistence of Cyc8 inclusions. To decipher this, we monitored Cyc8 inclusions by fluorescence microscopy on parent or *gpd1Δgpd2Δ* cells expressing Cyc8-GFP after washing out the reversible transcription inhibitor PHN (Fig 6A). Yeast cells were treated with PHN for 5 minutes to inhibit transcription. As previously seen, PHN treatment was insufficient to induce significant amounts of Cyc8 inclusion formation alone (Fig 6B). After PHN treatment, cells were subsequently challenged with 1.2M sorbitol for 30 minutes to form Cyc8 inclusions, then the cells were moved to media containing 1.2M sorbitol with or without PHN. Cyc8 inclusions disappeared over the course of 60 minutes in parent cells after removal of PHN, but were maintained up to 120 minutes in *gpd1Δgpd2Δ* cells after removal of PHN. Cyc8 inclusions did not resolve in either parent or *gpd1Δgpd2Δ* cells when PHN was maintained in the media (no washout). Quantification of the total number of cells displaying Cyc8 inclusions following drug removal showed a significant loss of Cyc8 inclusions only in parent cells by 30 minutes, reaching basal levels by 120 minutes after removal of PHN (Fig 6C). Cells incapable of producing glycerol, however, showed no resolution of Cyc8 inclusions.

Under the same experimental design, we monitored intracellular glycerol content of parent or *gpd1Δgpd2Δ* cells after removal of PHN (Fig 6D). As expected, parent cells showed a significant accumulation of intracellular glycerol following removal of PHN, which was inversely correlated to the amount of Cyc8 inclusions seen previously. In line with this, parent cells had greatly reduced glycerol accumulation when PHN was maintained in the media. Cells that were *gpd1Δgpd2Δ* showed virtually no intracellular glycerol, regardless of the presence of PHN.

Finally, we tracked Cyc8 SUMOylation in parent or *gpd1Δgpd2Δ cells* after transcriptional restart (Fig 6E). As we showed in Fig 2, transcription inhibition was insufficient to induce Cyc8 SUMOylation in either strain prior to the onset of stress. Application of the hyperosmotic stressor initiated Cyc8 SUMOylation in both parent and *gpd1Δgpd2Δ* cells as observed after 30 minutes of exposure. As expected, maintenance of transcriptional blockade resulted in sustained Cyc8 SUMOylation, regardless of the presence of glycerol biogenesis enzymes (Fig 6E, no wash). In parent cells, removal of transcriptional block (Fig 6E, wash) resulted in deSUMOylation of Cyc8, tightly correlating with the rate of glycerol accumulation. In *gpd1Δgpd2Δ* cells, however, Cyc8 SUMOylation was maintained even upon removal of PHN. Altogether, these results support the idea that accumulation of the osmolyte glycerol is the necessary signal for the resolution of Cyc8 inclusions and Cyc8 deSUMOylation during exposure to hyperosmotic stress.

## Discussion

**Fig 7:**
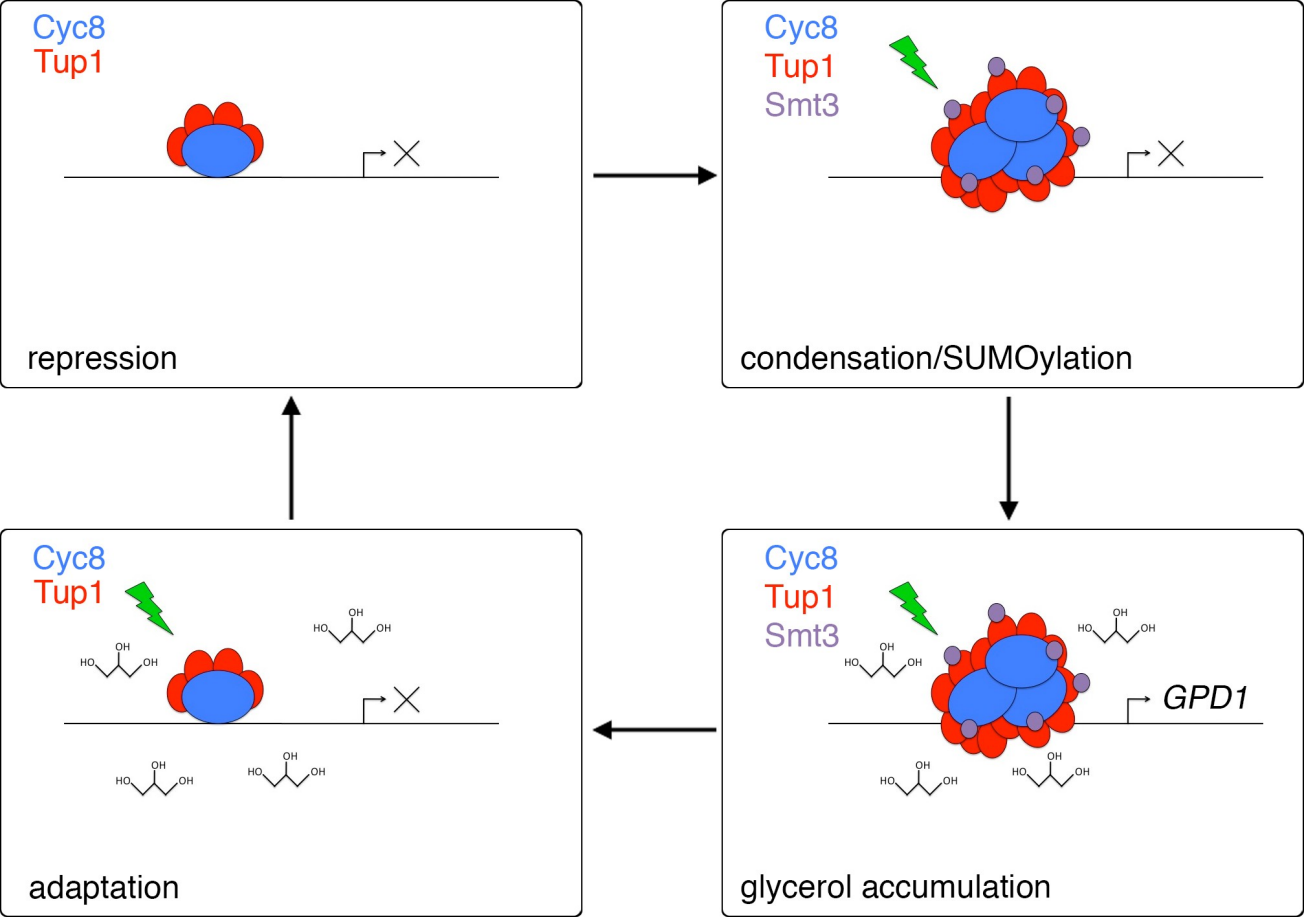
A model for the interplay of Cyc8 SUMOylation and inclusion formation during adaptation to hyperosmotic stress. Under iso-osmotic conditions, the Cyc8-Tup1 corepressor complex is diffusely localized across the nucleus, facilitating repression of target genes. Hyperosmotic stress induces rapid assembly of Cyc8-Tup1 inclusions and SUMOylation of the complex, which assists in orienting the complex at the promoter of specific genes like *GPD1*. Expression of *GPD1* and subsequent glycerol accumulation facilitates adaptation to hyperosmotic stress, upon which Cyc8-Tup1 are deSUMOylated and inclusions are resolved.

Cellular stress responses involve integration of multiple signaling pathways to adapt the cell’s metabolism to the stress. Here, we examined the integration of signaling pathways during hyperosmotic stress and reveal a novel intersection between Hog1 MAPK-dependent phosphorylation and SUMOylation of a key transcription corepressor complex Cyc8-Tup1. In turn, these signaling events work in concert to orchestrate spatiotemporal regulation of the Cyc8-Tup1 complex, which undergoes a phase transition to concentrate at specific promoters. These biochemical and cell biological processes are necessary for timely and efficient expression of survival genes, namely those responsible for the production of the key osmolyte glycerol, whose intracellular accumulation provides a feedback loop needed to reset the system following the adaptive process. We summarize these observations in Fig 7.

Environmental stressors are diverse and each stress inhibits cellular functions in unique ways. Cellular stressors can rapidly cause irreparable damage to biomolecules and cellular structures, so it is paramount that cells respond to stress with expediency and efficiency. As such, organisms have evolved fast biophysical mechanisms to expedite slower biochemical responses during adaptation, as seen with the yeast proteins Pab1 and Sup35 that condense into focal structures during stress conditions [15, 29]. Hyperosmotic stress causes significant water efflux from cells, decreasing the total cellular volume and increasing macromolecular crowding [30], which has been implicated as an important factor in the condensation of protein-RNA complexes into phase separated droplets *in vitro* [31]. Moreover, it is well documented that hyperosmotic stress drives the formation of various cytoplasmic membraneless organelles, including stress granules in yeast and P granules in *Caenorhabditis elegans* [32, 33]. These dynamic bodies are primarily composed of proteins rich in low complexity sequences that are particularly enriched in polar residues such as asparagine and glutamine [34]. Cyc8 contains a glutamine-rich prion domain that we have previously shown significantly contributes to Cyc8 inclusion formation [10]. The data presented here are consistent with a model that the Cyc8 inclusions are membraneless organelles that form under stress conditions, with control of Cyc8 inclusions regulated in part through SUMOylation and osmolyte concentration.

In accord with this model, we found that glycerol biosynthesis was a key regulator of the persistence of Cyc8 inclusions, as their lifetime was inversely correlated with cellular glycerol content after exposure to hyperosmotic stress. Compatible osmolytes have been implicated as regulators of cytoplasmic inclusions during osmotic stress in multiple organisms [32, 33]. Yeast cells accumulate glycerol following exposure to hyperosmotic stress to reestablish ionic balance, retain water, and counteract molecular crowding [35]. Our data suggest that intracellular osmotic conditions modulate Cyc8 inclusion coalescence and dissolution and concomitant SUMOylation and deSUMOylation.

However, we note that there are non-mutually exclusive alternative models that could also account for the observations presented here. Glycerol is capable of preventing protein aggregation *in vitro* by altering protein-solvent interactions and promoting a larger radius of hydration [36]. In addition, glycerol has been shown to act as a chemical chaperone that promotes the native folding state in proteins susceptible to misfolding [37]. In line with these previous observations, we observe Cyc8 inclusions forming only upon onset of hyperosmotic stress, and resolving only after sufficient accumulation of intracellular glycerol. It is formally possible that glycerol acts as a chemical chaperone to dissolve Cyc8 inclusions; however, our prior studies on Cyc8 inclusions do not show phenomena consistent with stable protein aggregation [10]. Moreover, chemical chaperones are hypothesized to promote native folding, not by direct binding, but rather by stabilizing a protein’s hydration shell [38]. Therefore, we think it is unlikely that glycerol is a direct ligand for Cyc8, but rather facilitates inclusion dissolution by rehydrating the nucleoplasm and reducing macromolecular crowding in the organelle.

Mechanistically, it is unclear whether the formation and subsequent resolution of Cyc8 inclusions occurs spontaneously or is mediated by some as yet unidentified factor. While widespread kinase inhibition by pretreatment with the broad-spectrum kinase inhibitor staurosporine did not change Cyc8 SUMOylation dynamics during adaptation to hyperosmotic stress (S3 Fig), it is possible that some other factor not observed in these studies guides Cyc8 into and out of inclusions. In turn, glycerol accumulation may affect some other signaling pathway that functions to modulate Cyc8 inclusion dynamics. Deciphering further the biophysical and signaling contributions of glycerol effects on Cyc8 dynamic SUMOylation and inclusion formation will be a topic of future study.

In the larger context of transcription, previous work has demonstrated that SUMO and the SUMOylation machinery are enriched at the promoters of inducible genes, and both Tup1 and Cyc8 SUMOylation have been implicated in controlling the timing of inducible genes [39–41]. A wide variety of transcriptional/translational modulators are known to be SUMOylated, and the consequences of these SUMOylation events are equally diverse. Similar to our observations with Cyc8, some of these SUMO substrates are enriched in nuclear inclusions or have prion-like propensities for aggregation, including *Arabidopsis* TCP proteins and human CPEB3 [42, 43]. Also, recent advances in super-resolution microscopy and gene editing have shown a well-characterized binding partner of Cyc8 –the Mediator complex–undergoing phase-separation at inducible loci in a transcription-dependent manner [44, 45]. Given this, it is plausible that biophysically driven inclusion formation of aggregation-prone transcription and translation factors is thematic throughout nature, and occurs in response to diverse stimuli. Once initial scaffolds are constructed, post-translational modifications like SUMOylation allow for the potential recruitment of necessary interactors or regulate the duration of the inclusions to elicit the appropriate temporal biological responses (such as the recruitment of transcriptional machinery or chromatin modifiers). Our work provides new insights on genetic and metabolic factors that can regulate the stress-induced formation of dynamic inclusion bodies in transcription factors, and may shed new light on the interplay of different post-translational modifications during the temporal adaptation to stress.

## Methods

### Yeast strains and plasmids

Yeast strains and plasmids used in this study are listed in Tables 1 and 2. Standard yeast genetic methods were used for these studies [46]. All gene deletions were verified by colony PCR and phenotypic analyses when available.

**Table 1:** Yeast strains.

Strain	Genotype	Reference
RGY5266	met15*Δ*0, his3*Δ*1, ura3*Δ*0, leu2*Δ*0, 6His-FLAG-SMT3::HIS3MX6	[10]
RGY5645	met15*Δ*0, his3*Δ*1, ura3*Δ*0, leu2*Δ*0, 6His-FLAG-SMT3::HIS3MX6, cyc8*Δ*	[10]
RGY5654	met15*Δ*0, his3*Δ*1, ura3*Δ*0, leu2*Δ*0, 6His-FLAG-SMT3::HIS3MX6, hog1*Δ*	[10]
RGY5708	met15*Δ*0, his3*Δ*1, ura3*Δ*0, leu2*Δ*0, 6His-FLAG-SMT3::HIS3MX6, tup1*Δ*::TUP1-3HA::URA3	[10]
RGY5809	met15*Δ*0, his3*Δ*1, ura3*Δ*0, leu2*Δ*0, 6His-FLAG-SMT3::HIS3MX6, tup1*Δ*::TUP1-3HA::URA3, hog1*Δ*	this study
RGY5820	met15*Δ*0, his3*Δ*1, ura3*Δ*0, leu2*Δ*0, 6His-FLAG-SMT3::HIS3MX6, cyc8*Δ*::CYC8-eGFP::LEU2	[10]
RGY5822	met15*Δ*0, his3*Δ*1, ura3*Δ*0, leu2*Δ*0, 6His-FLAG-SMT3::HIS3MX6, cyc8*Δ*::CYC8(K735R,K736R,K738R,K748R)-eGFP::LEU2	[10]
RGY5824	met15*Δ*0, his3*Δ*1, ura3*Δ*0, leu2*Δ*0, 6His-FLAG-SMT3::HIS3MX6, cyc8*Δ*::CYC8-3HSV::LEU2	[10]
RGY5825	met15*Δ*0, his3*Δ*1, ura3*Δ*0, leu2*Δ*0, 6His-FLAG-SMT3::HIS3MX6, cyc8*Δ*::CYC8(K735R,K736R,K738R,K748R)-3HSV::LEU2	[10]
RGY5855	met15*Δ*0, his3*Δ*1, ura3*Δ*0, leu2*Δ*0, 6His-FLAG-SMT3::HIS3MX6, gpd1*Δ*	this study
RGY5859	met15*Δ*0, his3*Δ*1, ura3*Δ*0, leu2*Δ*0, 6His-FLAG-SMT3::HIS3MX6, gpp2*Δ*	this study
RGY5862	met15*Δ*0, his3*Δ*1, ura3*Δ*0, leu2*Δ*0, 6His-FLAG-SMT3::HIS3MX6, gpd1*Δ*, gpd2*Δ*	this study
RGY5863	met15*Δ*0, his3*Δ*1, ura3*Δ*0, leu2*Δ*0, 6His-FLAG-SMT3::HIS3MX6, gpd2*Δ*	this study
RGY5913	met15*Δ*0, his3*Δ*1, ura3*ΔΔ*0, leu2*Δ*0, 6His-FLAG-SMT3::HIS3MX6, gpp1*Δ*, gpp2*Δ*	this study
RGY5921	met15*Δ*0, his3*Δ*1, ura3*Δ*0, leu2*Δ*0, 6His-FLAG-SMT3::HIS3MX6, gpp2*Δ*	this study
RGY5961	met15*Δ*0, his3*Δ*1, ura3*Δ*0, leu2*Δ*0, 6His-FLAG-SMT3::HIS3MX6, ste11*Δ*	this study
RGY5962	met15*Δ*0, his3*Δ*1, ura3*Δ*0, leu2*Δ*0, 6His-FLAG-SMT3::HIS3MX6, cyc8*Δ*, hog1*Δ*	this study
RGY5988	met15*Δ*0, his3*Δ*1, ura3*Δ*0, leu2*Δ*0, 6His-FLAG-SMT3::HIS3MX6, cyc8*Δ*::CYC8-eGFP::LEU2, hog1*Δ*	this study
RGY5989	met15*Δ*0, his3*ΔΔ*1, ura3*Δ*0, leu2*Δ*0, 6His-FLAG-SMT3::HIS3MX6, cyc8*Δ*::CYC8(K735R,K736R,K738R,K748R)-eGFP::LEU2, hog1*Δ*	this study
RGY5990	met15*Δ*0, his3*Δ*1, ura3*Δ*0, leu2*Δ*0, 6His-FLAG-SMT3::HIS3MX6, cyc8*Δ*::CYC8-3HSV::LEU2, hog1*Δ*	this study
RGY5991	met15*Δ*0, his3*Δ*1, ura3*Δ*0, leu2*Δ*0, 6His-FLAG-SMT3::HIS3MX6, cyc8*Δ*::CYC8(K735R,K736R,K738R,K748R)-3HSV::LEU2, hog1*Δ*	this study
RGY5996	met15*Δ*0, his3*Δ*1, ura3*Δ*0, leu2*Δ*0, 6His-FLAG-SMT3::HIS3MX6, cyc8*Δ*::CYC8-eGFP::LEU2, gpd1*Δ*, gpd2*Δ*	this study
RGY5997	met15*Δ*0, his3*Δ*1, ura3*Δ*0, leu2*Δ*0, 6His-FLAG-SMT3::HIS3MX6, cyc8*Δ*::CYC8(K735R,K736R,K738R,K748R)-eGFP::LEU2, gpd1*Δ*, gpd2*Δ*	this study
RGY5998	met15*Δ*0, his3*Δ*1, ura3*Δ*0, leu2*Δ*0, 6His-FLAG-SMT3::HIS3MX6, cyc8*Δ*::CYC8-3HSV::LEU2, gpd1*Δ*, gpd2*Δ*	this study
RGY5999	met15*Δ*0, his3*Δ*1, ura3*Δ*0, leu2*Δ*0, 6His-FLAG-SMT3::HIS3MX6, cyc8*Δ*::CYC8(K735R,K736R,K738R,K748R)-3HSV::LEU2, gpd1*Δ*, gpd2*Δ*	this study

**Table 2:** Yeast plasmids.

Plasmid	Encoded protein	Parent vector	Reference
pRG4059	*TUP1-3HA*	pRS406	[10]
pRG4084	*CYC8-3HSV*	pRS405	[10]
pRG4085	*CYC8-eGFP*	pRS405	[10]
pRG4113	*CYC8(K735R*,*K736R*,*K738R*,*K748R)-3HSV*	pRS405	[10]
pRG4175	*HOG1*	pRS416	this study
pRG4186	*HOG1-T174A/Y176F*	pRS416	this study
pRG4188	*HOG1-D144A*	pRS416	this study

### Growth and stress conditions

Cells were grown to a density of ~1.5x10^7^ cells/ml at 30°C in yeast complete (YC) media prior to stress induction. All 0 time point samples were collected before stress induction. For induction of hyperosmotic stress, equal volumes of culture and YC+2.4M sorbitol were combined for a final concentration of 1.2M sorbitol. For transcription inhibition, cells were treated at room temperature for 5 minutes with either 4ug/ml thiolutin (Sigma Aldrich), 500ug/ml 1,10-phenanthroline (Sigma Aldrich), or an equal volume of DMSO vehicle control prior to the induction of hyperosmotic stress. For broad-spectrum kinase inhibition, cells were treated with staurosporine at the indicated concentrations or an equal volume of DMSO at 30°C for 1 hour prior to the induction of hyperosmotic stress.

### SUMOylated protein purification

50ml aliquots of cells were collected at each time point after stress and flash frozen in liquid nitrogen. Harvested cells were lysed by vortexing with glass beads at 4°C in 1ml denaturing lysis buffer (8M urea, 50mM Tris pH 8.0, 0.05% SDS with 2mM PMSF and 20mM NEM). An aliquot representing 5% of the input was set aside. Cell lysates were incubated with TALON resin (Novagen) overnight at 4°C. The resin was washed 3x with wash buffer (8M urea, 50mM Tris pH 8.0, 200mM NaCl, 0.05% SDS, 5mM imidazole). SUMOylated proteins were eluted from the column by addition of loading buffer (8M urea, 10mM MOPS, 10mM EDTA, 1% SDS, 0.01% bromophenol blue, pH 6.8) and incubation at 65°C for 10 minutes.

### Western analysis

SUMOylated proteins were resolved by SDS-PAGE using 4–20% gradient gels. Western analyses were performed with mouse anti-FLAG (1:2500, Sigma), mouse anti-HSV (1:2500, Novagen), mouse anti-HA (1:2500, Sigma), mouse anti-actin (1:2500, Abcam), rabbit anti PKA consensus phospho-motif (RRXS*/T*) (1:2500, CST), or rabbit phospho-p38 (1:2500, CST).

### Chromatin immunoprecipitation

Chromatin immunoprecipitation was performed as previously described [47]. Briefly, 50ml aliquots of cells were grown to a density of ~1.5x10^7^ cells/ml at 30°C in rich medium prior to stress induction. A pre-stress sample was collected prior to induction of stress. Hyperosmotic stress was induced by addition of sorbitol to a final concentration of 1.2M, and samples were collected at the indicated times. Protein-DNA crosslinking was initiated by addition of formaldehyde to a concentration of 1% and incubated at room temperature for 15 minutes with continuous swirling. The reaction was quenched by addition of glycine to 0.125M for 5 minutes at room temperature. Samples were washed in 1X TBS, collected by centrifugation, and flash frozen in liquid nitrogen. Cells were lysed by bead-beating in 5ml breaking buffer (100mM Tris pH 7.9, 20% glycerol, 2mM PMSF) at 4°C. Insoluble chromatin was collected by centrifugation and resuspended in 700uL ChIP lysis buffer (50mM HEPES pH 7.5, 140mM NaCl, 1% Triton X-100, 0.1% deoxycholate, 2mM PMSF) and sonicated to shear chromatin. A sample prior to immunoprecipitation was collected to represent input controls. Immunoprecipitation was performed on 200ul sheared chromatin by incubation with mouse anti-HSV bound to protein A-sepharose beads overnight at 4°C. Beads were washed twice each with ChIP lysis buffer, high salt ChIP lysis buffer (50mM HEPES pH 7.5, 500mM NaCl, 1% Triton X-100, 0.1% deoxycholate, 2mM PMSF), ChIP wash buffer (10mM Tris pH 8.0, 250mM LiCl, 0.5% NP-40, 0.5% deoxycholate, 1mM EDTA, 2mM PMSF), and 1X TE buffer. Immunoprecipitated protein-DNA complexes were eluted by incubation in ChIP elution buffer (50mM Tris pH 8.0, 1% SDS, 10mM EDTA) at 65°C for 10 minutes. Samples were treated Proteinase K (New England Biolabs) and RNase at 50°C for one hour, followed by removal of crosslinking by incubation at 65C overnight. Input and elute DNA was isolated using QIAprep spin columns (Qiagen) and analyzed by qPCR.

### qPCR analyses

Quantitative real-time PCR was performed using a 7500 Fast Real-Time PCR system (Applied Biosystems). Reactions were performed on ChIP inputs and eluates using PowerUP SYBR Green Master Mix (Applied Biosystems) with specific primers for the *GPD1* UAS or *ACT1* control. *Δ*Ct values were calculated for each condition and corrected by their respective *ACT1 Δ*Ct. Results were converted to *ΔΔ*Ct and normalized to each cell’s respective pre-stress time point. Primer sequences for *GPD1*: 5’–TCTCACCTCTCACCGCTGAC– 3’, 5’–AGACTTGCTCAAACCCCAGGAG– 3’. Primer sequences for *ACT1*: 5’–TGGCCGGTAGAGATTTGACTGACT– 3’, 5’–TCGAAGTAAGGCGACGTAACAT– 3’.

### Glycerol accumulation assays

10ml aliquots of cells were grown to a density of ~1.5x10^7^ cells/ml at 30°C in rich medium. A pre-stress sample was collected prior to induction of stress. Hyperosmotic stress was induced by addition of sorbitol to a final concentration of 1.2M, and samples were collected at the indicated time points. Cells were collected by centrifugation, resuspended in 100uL 1X TBS, and incubated at 95°C for 10 minutes. Supernatant was collected by centrifugation and glycerol concentration in the resulting fraction was measured using a commercial enzymatic assay kit (Sigma Aldrich).

### Fluorescence microscopy

Aliquots of cells at each time point after hyperosmotic stress were removed, fixed in 4% paraformaldehyde solution for 15 minutes at room temperature and then washed with PBS. Cells were imaged on a Nikon Eclipse 90i with a100X objective, filters for GFP (HC HiSN 0 Shift filter set with excitation wavelength (450–490 nm), dichroic mirror (495 nm), and emission filter (500–550 nm)), and a Photometrics Cool Snap HQ2 cooled CCD camera with NIS-Elements acquisition software.

### Image processing

All blots were scanned using an Epson Perfection V350 Photo scanner at 300 dpi. All images were processed with a Mac iMac or Pro computer (Apple) using Photoshop CS or CS4 (Adobe).

## Supporting information

S1 Fig Confirmation of osmosensitivity of *hog1* mutants and comparison of global SUMOylation in *ste11Δ* vs. *hog1Δ*. (A) Spot titer assay confirming osmosensitivity of various Hog1 mutants. Parent or *hog1Δ* cells expressing the indicated constructs were spotted in ten-fold serial dilutions on the indicated medias and grown at 30C for two days. (B) Prolonged Cyc8 SUMOylation in the absence of Hog1 is not an artifact of crosstalk. Parent, *ste11Δ*, or *hog1Δ* cells expressing 6His-FLAG-SMT3 were treated with 1.2M sorbitol and collected at the indicated time points. Whole cell extracts were separated by SDS-PAGE and Western analysis with anti-FLAG antibodies to identify SUMOylated proteins. Anti-PGK1 antibodies were used to detect *PGK1* as a loading control.
(TIF)

S2 Fig Deletion of glycerol biosynthesis genes or transcriptional blockade inhibit glycerol accumulation during hyperosmotic stress. (A) Confirmation of reduced glycerol content in glycerol biosynthetic mutants. Indicated cells were analyzed by glycerol assay as described in Fig 4B. Error bars show SD. (B) Transcription inhibitors slow glycerol accumulation during hyperosmotic stress. Parent cells were grown in triplicate in rich medium and treated with the indicated compound for 5 minutes at room temperature and hyperosmotic stress was initiated by addition of sorbitol to 1.2M. Cells were collected and glycerol content was analyzed as described in Fig 4B. Error bars show SD.
(TIF)

S3 Fig Regulation of Cyc8-SUMOylation during adaptation to hyperosmotic stress is not affected by targets of the broad-spectrum kinase inhibitor staurosporine. (A). At 1 μM staurosporine, PKA is inhibited, while Hog1 is not. Cells of the indicated genotypes were incubated with the indicated concentrations of staurosporine or an equal volume of DMSO for 1 hour, then treated with 1.2 M sorbitol for 15 min. Cells were collected, lysed, and subjected to Western analysis for the phosphorylated PKA motif, autophosphorylated Hog1, and Pgk1 as a loading control. (B) Cyc8 deSUMOylation kinetics are not affected by kinase activity. Indicated cells were incubated with 1 μM staurosporine or an equal volume of DMSO for 1 hour, then treated, collected, and analyzed as described in Fig 1A. Cyc8 was identified by Western analysis using anti-HSV antibodies. Total Cyc8 in the input fraction was used as a loading control, while anti-pP38 was used as a control for Hog1 activation.
(TIF)
