# Supplementary figures and images for: Osmolyte accumulation regulates the SUMOylation and inclusion dynamics of the prionogenic Cyc8-Tup1 transcription corepressor
Source: PLoS Genet. 2019 Apr 22;15(4):e1008115. doi: 10.1371/journal.pgen.1008115 (PMC6497323; doi:10.1371/journal.pgen.1008115)

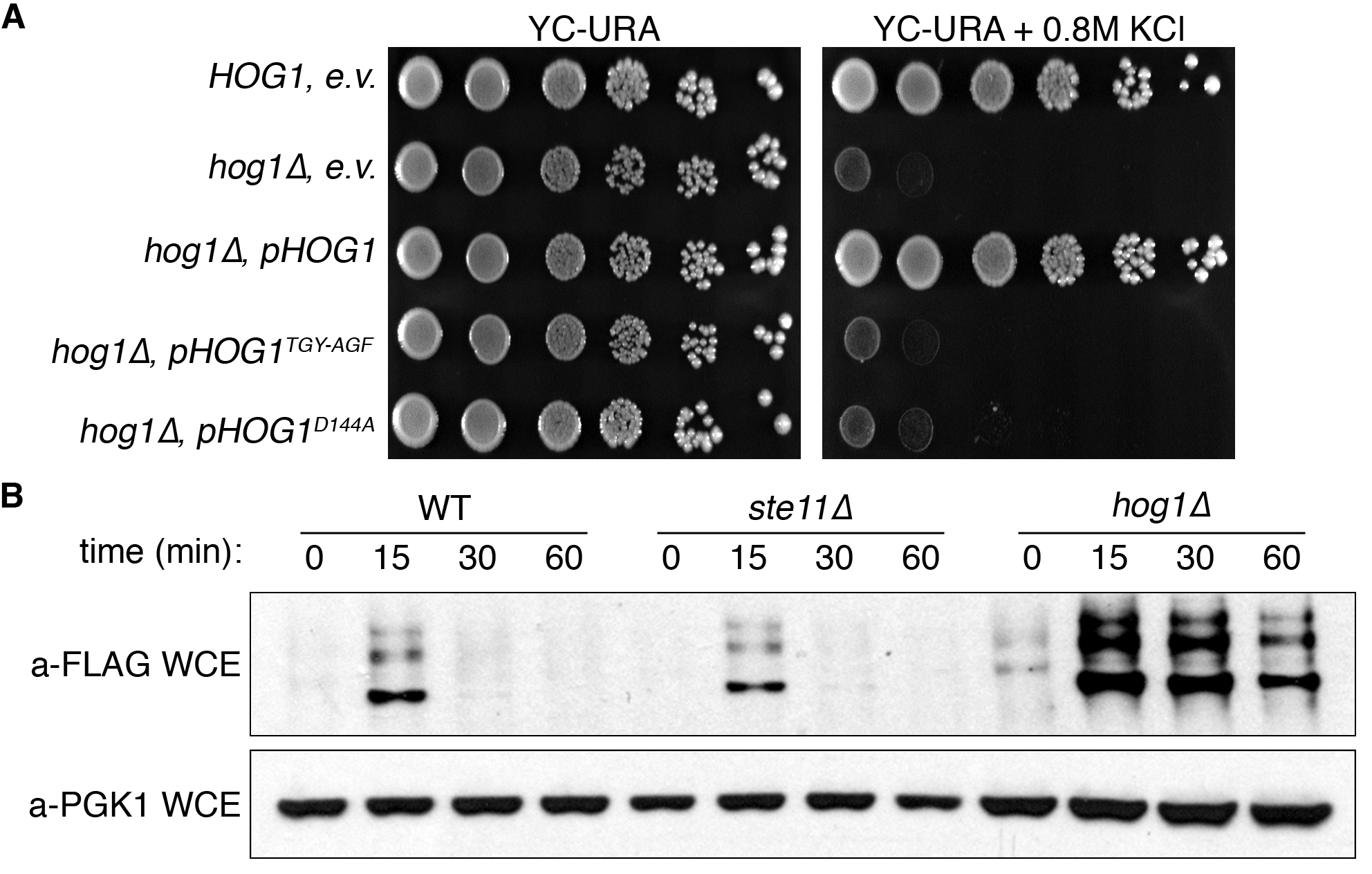

Supplement: S1 Fig — (A) Spot titer assay confirming osmosensitivity of various Hog1 mutants. Parent or hog1Δ cells expressing the indicated constructs were spotted in ten-fold serial dilutions on the indicated medias and grown at 30C for two days. (B) Prolonged Cyc8 SUMOylation in the absence of Hog1 is not an artifact of crosstalk. Parent, ste11Δ, or hog1Δ cells expressing 6His-FLAG-SMT3 were treated with 1.2M sorbitol and collected at the indicated time points. Whole cell extracts were separated by SDS-PAGE and Western analysis with anti-FLAG antibodies to identify SUMOylated proteins. Anti-PGK1 antibodies were used to detect PGK1 as a loading control. (TIF) [file pgen.1008115.s001.tif]

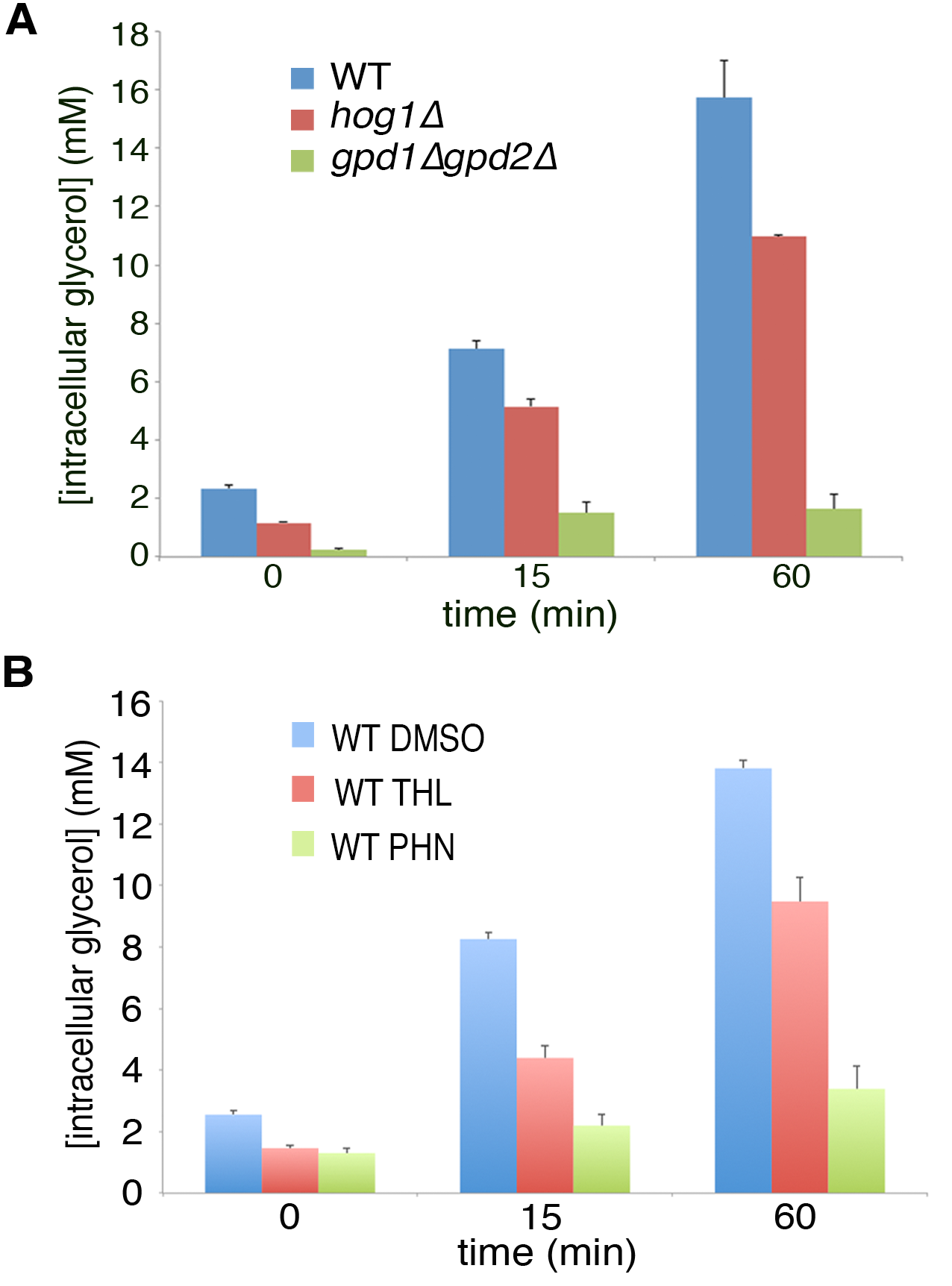

Supplement: S2 Fig — (A) Confirmation of reduced glycerol content in glycerol biosynthetic mutants. Indicated cells were analyzed by glycerol assay as described in Fig 4B. Error bars show SD. (B) Transcription inhibitors slow glycerol accumulation during hyperosmotic stress. Parent cells were grown in triplicate in rich medium and treated with the indicated compound for 5 minutes at room temperature and hyperosmotic stress was initiated by addition of sorbitol to 1.2M. Cells were collected and glycerol content was analyzed as described in Fig 4B. Error bars show SD. (TIF) [file pgen.1008115.s002.tif]

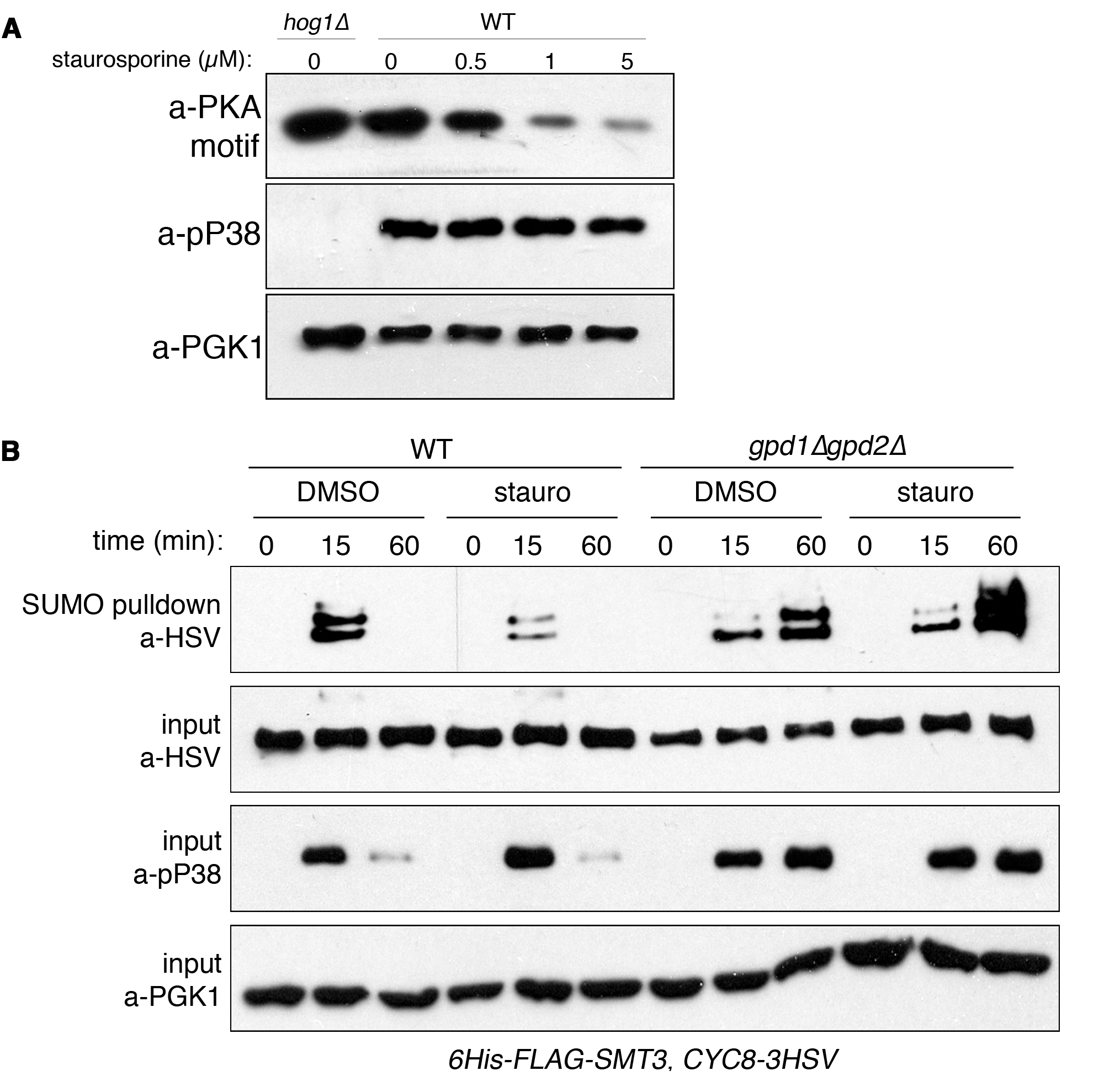

Supplement: S3 Fig — (A). At 1 μM staurosporine, PKA is inhibited, while Hog1 is not. Cells of the indicated genotypes were incubated with the indicated concentrations of staurosporine or an equal volume of DMSO for 1 hour, then treated with 1.2 M sorbitol for 15 min. Cells were collected, lysed, and subjected to Western analysis for the phosphorylated PKA motif, autophosphorylated Hog1, and Pgk1 as a loading control. (B) Cyc8 deSUMOylation kinetics are not affected by kinase activity. Indicated cells were incubated with 1 μM staurosporine or an equal volume of DMSO for 1 hour, then treated, collected, and analyzed as described in Fig 1A. Cyc8 was identified by Western analysis using anti-HSV antibodies. Total Cyc8 in the input fraction was used as a loading control, while anti-pP38 was used as a control for Hog1 activation. (TIF) [file pgen.1008115.s003.tif]
